# A Web Portal for Communicating Polygenic Risk Score Results for Health Care Use—The P5 Study

**DOI:** 10.3389/fgene.2021.763159

**Published:** 2021-10-29

**Authors:** Heidi Marjonen, Minttu Marttila, Teemu Paajanen, Marleena Vornanen, Minna Brunfeldt, Anni Joensuu, Otto Halmesvaara, Kimmo Aro, Mervi Alanne-Kinnunen, Pekka Jousilahti, Katja Borodulin, Seppo Koskinen, Tiinamaija Tuomi, Pirjo Ilanne-Parikka, Jaana Lindström, Merja K. Laine, Kirsi Auro, Helena Kääriäinen, Markus Perola, Kati Kristiansson

**Affiliations:** ^1^ Department of Public Health and Welfare, Finnish Institute for Health and Welfare, Helsinki, Finland; ^2^ Research Program for Clinical and Molecular Metabolism, Faculty of Medicine, University of Helsinki, Helsinki, Finland; ^3^ Department of Social Research, Faculty of Social Sciences, University of Helsinki, Helsinki, Finland; ^4^ Negen Ltd., Helsinki, Finland; ^5^ Age Institute, Helsinki, Finland; ^6^ Abdominal Centre, Endocrinology, Helsinki University Hospital, Helsinki, Finland; ^7^ Folkhälsan Research Centre, Helsinki, Finland; ^8^ Institute for Molecular Medicine Finland, Helsinki, Finland; ^9^ Lund University Diabetes Centre, Lund University, Malmö, Sweden; ^10^ Finnish Diabetes Association, Tampere, Finland; ^11^ Department of General Practice and Primary Health Care, University of Helsinki and Helsinki University Hospital, Helsinki, Finland

**Keywords:** web portal, type 2 diabetes, coronary heart disease, venous thromboembolism, polygenic risk sore, finhealth 2017 study, P5 study, precision population health

## Abstract

We present a method for communicating personalized genetic risk information to citizens and their physicians using a secure web portal. We apply the method for 3,177 Finnish individuals in the P5 Study where estimates of genetic and absolute risk, based on genetic and clinical risk factors, of future disease are reported to study participants, allowing individuals to participate in managing their own health. Our method facilitates using polygenic risk score as a personalized tool to estimate a person’s future disease risk while offering a way for health care professionals to utilize the polygenic risk scores as a preventive tool in patient care.

## Introduction

The increasing accessibility and level of media coverage of direct-to-consumer (DTC) genomic testing provided by private companies has raised interest among individuals to receive and understand detailed information about their genetic background and its consequences for their health (Horton et al., 2019). At the same time, national genome strategies that are designed to utilise genetics in public health care are being introduced globally (Stark et al., 2019). The DTC tests already present a new challenge for professionals in public health services, since the interpretation of these genomic test results is often requested from the general practitioners working in everyday patient care. Systematic use of genetic tests for common diseases in health care would further amplify the need for new methods and guidelines in the field.

In several countries, there are established processes for testing rare genetic variants with known clinical implications, single clinical variants (SCVs), and the test results and genetic counselling are provided in specialised clinics. As genetic testing for multiple variants in the area of common complex diseases and traits becomes increasingly widespread the specialised clinics can no longer support interpretation of all results the citizens receive. Thus there is a need for proof of concept studies on how genetic risk reports with information on common diseases could be delivered to both patients and their physicians who have no broad training in genetics. Interpretation of the genetic results, as well as instructions on how to implement them in clinical work to maximize the impact for patient care, need to be determined. While face-to-face genetic counselling for each citizen is unrealistic, the return of genetic information in the forms of polygenic risk scores (PRS) and SCVs and related lifestyle advice should be accessible, personalised, and comprehensible for the receiving individual. Here, feasible digital solutions that could offer a way to implement above mentioned interventions for a large number of participants at once but still maintaining one’s privacy are needed.

Recent advances in whole genome genotyping have offered a feasible way to utilise genetic information in health care. PRS is a single value that measures an individual’s genetic burden to a disorder or trait. It is based on genome-wide association study (GWAS) summary statistics and can be calculated in several ways (Choi et al., 2020). Since nowadays genotyping chip arrays are cheap and an individual’s genome remains constant throughout their life, genotyping does not have to be done repeatedly, making PRS a very tempting and cost-effective tool for clinical implementation. In research studies, PRS has suggested benefits in patient screening, stratification of disease subtypes, prediction of future disease risk, and the disentangling of the heterogeneity of cardiometabolic disease endophenotypes (Maas et al., 2016; Natarajan et al., 2017).

This paper introduces a method for sharing both PRS and SCV information in public health care for everyday clinical practice. We derived population-specific PRSs and returned information on individual genomic risk and estimates of future 10-years disease risk based on both genetic and traditional risk factors to 3,177 Finnish biobank participants. We demonstrate how the information can be reported simultaneously to a large number of individuals and, when needed, to their physicians with a “doctor’s note” using a secure internet portal taking into account General Data Protection Regulations. We tested the process with type 2 diabetes (T2D), coronary heart disease (CHD), and venous thrombosis (VTE), due to the major burden they pose for the health care system in Finland, and because recent global research results have shown the benefit of PRS in T2D and CHD risk prediction (Khera et al., 2018; Mars et al., 2020). In addition to sharing genetic information, our portal method also provided a possibility to start a randomized control trial (RCT) where the participant’s reactions to genetic risk information as well as effects on health behaviour can be examined in a separate follow-up study. This paper presents the process of portal construction and returning the results to participants in a RCT by using T2D as an example.

## Methods

### The Web Portal Infrastructure

The General Data Protection Regulation (GDPR) came into force on May 25, 2018 advocating the safe use and sharing of sensitive information, such as health, and genomic data. We set up a web portal to return genetic risk information to study participants in a high security environment. The data base instance where the sensitive information is stored is located within our institute’s (Finnish Institute for Health and Welfare) internal network, in the ePouta platform provided by CSC—IT CENTER FOR SCIENCE LTD (CSC-IT Center for Science Ltd, 2021a). ePouta is based on the open source cloud software called OpenStack. From ePouta, access endpoint was configured through firewall utilizing encrypted point-to-point tunnel to another CSC provided instance, cPouta (CSC-IT Center for Science Ltd, 2021b). CPouta harbors a web server interface which allows SSL-encrypted traffic to and from internet, the MyP5 web portal interface.

### Portal User Identification Set Up

To ensure compliance with GDPR, the e-services of public administration organizations in Finland require strong identification where the user’s identity can be verified. We linked our results portal interface with such identification method, the Suomi. fi e-Identification (Suomi.fi e-Identification, 2021). Every time our study participant goes to the portal website, the identification service activates and the participant identifies themselves using Finnish online banking codes, a certificate card or a mobile certificate before gaining access to the portal. The identification data is transmitted over a secure connection, and the process ensures the protection of privacy in accordance with the Finnish legislation and provisions on the processing of personal data.

### Recruitment of Participants for Portal Testing

The workflow for returning validated genetic risk and absolute disease risk information, including both genetic and traditional risk factors, to study participants and their physicians using the portal was tested in a research study called “P5”. The P5 Study updates the predictive, preventive, personalised, and participatory approach (P4 medicine) by adding a fifth P, population health. The study was approved by the Coordinating Ethics Committee of the Hospital District of Helsinki and Uusimaa (37/13/03/00/2016 section 55).

The P5 participants were recruited from a previously conducted study, The FinHealth 2017 Study, which collected health and well-being information of adults residing in Finland in 2017 (Borodulin and Sääksjärvi, 2019). The FinHealth 2017 Study was carried out at 50 localities with a random sample of 10,247 people over the age of 18. The FinHealth 2017 Study comprised a physical examination, laboratory measurements and questionnaires.

The P5 Study invited, via a letter, all FinHealth 2017 Study participants who had given a blood sample and consented voluntarily to THL biobank (N = 6,189) to take part in a new study. In this new P5 Study, a participant would find out one’s genomic predisposition for three diseases: T2D, CHD, and VTE. The P5 Study utilised health and genomic information obtained from the FinHealth 2017 Study to calculate the participants’ estimated overall risk for developing CHD, T2D, and VTE within the following 10 years. The study invitation was also sent to individuals who already had one or more of the studied diseases. The first invitation letter explained that (“you can still participate although you have one of the studied diseases”) and later the results provided through the portal separately for each of the diseases stated that (“if you already have this disease, then the risk assessment will no longer apply”). Since the risk information was provided through a web portal, a condition for participation was having internet access either with a computer or a mobile device. A total of 3,449 individual participated. The workflow of P5 Study is presented in Supplementary File S1.

### Creating Content for the Portal

#### Genome Data and the Polygenic Risk Score

The P5 study participants were whole-genome genotyped and imputed as part of the FinnGen study (FinnGen, 2021) by Affymetrix (Thermo Fisher Scientific, Santa Clara, CA, United States) (Axiom Genotyping chip). We used a previously published PRS by Khera et al. (2018). DNA polymorphisms with ambiguous strands (A/T or C/G) were removed from the score derivation and PRS was calculated with LDPred algorithm containing close to 7 million genomic variants for T2D and validated in the United Kingdom Biobank population. To calculate the PRS in the P5 Study, all variants were collected from the imputed data and weighted by their corresponding genotype effect sizes. Imputed data contained 94% of the original variants in the T2D PRS. After summing the variants together, the PRS was standardised using the mean and standard deviation (SD) of the PRS in an independent population sample (Supplementary File S2). If a variant was missing from the imputed data (missingness 0.05%), the population average frequency of the genotype was used in the calculation instead. A total of 3,177 P5 study participants had whole-genome genotyped and imputed data available.

#### Single Clinical Variants

In general, the GWAS used for PRS calculations rely on common gene variants, thus rare variants (Minor allele frequency (MAF) < 1%) that may be important for the heritability and manifestation of these diseases are often not included. For T2D, such rare variants are not included in disease risk evaluation in current clinical practices in Finland. For CHD and VTE, such variants exist: for VTE two mutations (F5 and F2) and for CHD seven FH mutations that increase the CHD risk are commonly tested in Finnish clinical practice if hereditary predisposition is suspected. Genotype data of P5 Study participants included the following SNPs: three LDL cholesterol-increasing LDLR variants FHTurku (NC_000019.10:g.11129654G >A), FHPori (NC_000019.10:g.11113293T >A) and FHPogosta (NC_000019.10:g.11116937G >A, MAF A = 0.0002 in Finland), the “Leiden” mutation in the *F5* gene (NC_000001.11:g.169549811C >T, MAF T = 0.02 in Finland) and a mutation in the *F2* (coagulation factor II) gene (NC_000011.10:g.46739505G >A, MAF A = 0.0046 in Finland), which are associated with thrombophilia, which increases the risk of having a venous thrombosis. We verified that the single clinical variants (SCVs) were successfully genotyped by a visual inspection of the genotyping clusters. The clusters were created by plotting allele signal intensity values of the variants from the genotyping chip data. The results portal was then used to report the carrier status of these SCVs as well as a relevant pharmacogenetic variant rs4149056 (NC_000012.12:g.21178615T >C, MAF C = 0.21 in Finland) of the *SLCO1B1* gene associated with LDL cholesterol-lowering medication for the P5 Study participants.

#### Estimating the Future Risk of Disease for P5 Study Participants

The analysis process of creating population based background data for genetic and absolute disease risk estimations in an independent FINRISK study cohort are presented in Supplementary File S2. As a result, T2D PRS associated in the Finnish FINRISK cohort HR: 1.5 per 1 sd increase, 95% CI: 1.43–1.63, *p*-value: <2*10–16.

In short, summary statistics from the selected FINRISK time-to-event model and baseline hazard were used to calculate a 10-years risk for developing T2D for each P5 participant using the predict () function in R. Since the baseline hazard is estimated non-parametrically in the Cox model, and thus the model’s coefficients are estimated without knowing the baseline hazard, the cumulative hazard rate was used to obtain the baseline hazard for the desired time period (10 years).

#### Allocation of Participants Into Groups for RCT

We randomised the P5 Study participants (N = 3,177) into two groups that received the risk information at different time points. Group One (N = 1,587) received a risk estimation based on both PRS and traditional risk factors for T2D and Group Two (N = 1,590) a risk estimation based on information of traditional risk factors only. After 2 months Group Two received the risk estimation based on both PRS and traditional risk factors for T2D. Before and after receiving the results, participants responded to questionnaires that assessed their reactions to the risk information, possible changes in their disease risk perceptions and health behaviour, and their opinions on whether the results were useful and clearly presented (for example: “Do you agree or disagree that the feedback you received on your results was difficult to understand?”). An example of the questionnaire is presented in Supplementary File S3. Questionnaire surveys will be continued to be held once a year for 5 years. The participants’ health data is followed up from national registers annually by the FinHealth 2017 Study.

#### Communicating the Risk Information Through a Website: “MyP5 Portal”

P5 Study participants received their personal disease risk estimates via a secure website, MyP5 portal. After receiving an email, text message, or a letter informing them about the availability of the results, they logged in to the MyP5-website (https://omap5.fi/en) using Suomi.fi e-Identification, which is a shared identification service for public administration e-services in Finland. On the website, each participant had access to their own 10-years absolute disease risk estimate of T2D, which was based on traditional risk factors (BMI, total cholesterol, HDL, systolic blood pressure, blood pressure-lowering medication, lipid-lowering medication, self-reported family history of T2D, smoking status), and PRS. The genetic risk (PRS) alone was provided as a single value and presented in relation to a normal distribution of the FINRISK Study population representing the general population in Finland. Furthermore, participants received a graph that presented their own total risk at baseline, the average risk of matched age population, as well as an estimate of the participant’s total risk at the age of 60 given the current traditional risk factor levels. In addition, the personal website included a risk calculator that enabled the participant to test the effects of changes in different lifestyle factors (BMI, blood pressure, and smoking) on the estimated risk.

#### Doctor’s Note

A health report “Doctor’s note” (Supplementary File S4) was provided with the risk estimate information to explain the results and to provide personalised instructions on how the participants can influence their disease risk with lifestyle modifications. The Doctor’s notes were created by a group of clinical experts with vast experience on clinical practise on the studied diseases. The clinical experts based their work on the Finnish Current Care Guidelines, which are independent, evidence-based clinical practice guidelines (https://www.kaypahoito.fi/en/). Participants received a tailored guidance for different T2D risk categories (<5%, 5–10%, 10–20%, >20%) and each age group (<50-years, 50–75-years, >75-years) to assist in the interpretation of the risk information in health care via the study participant. The Doctor’s note provided information on the implications of the results and a recommendation to contact health care if needed. Participants who were under 50 years old and had a 10-years T2D risk below 10% were guided with instructions on a healthy lifestyle and were told that they could share their results with their physician if they wanted to. Lifestyle instructions highlighted the importance of engaging in physical activity, to eat healthily and to maintain a normal weight. In addition, all participants were guided towards more information from the Finnish Diabetes Association website (https://www.diabetes.fi/en/finnish_diabetes_association). However, if the risk was over 10%, participants were encouraged (10–20% risk) or even recommended (>20% risk) to see a physician. In the 50–75 age group, the Doctor’s note was constructed in a similar way, although the participants with over a 10% risk were all recommended to see a physician. Since the FINRISK Study consisted of individuals aged 24–75 years, we were not able to calculate a 10-years risk for the P5 Study participants over the age of 75. For them the risk was provided as “your risk at the age of 75”. In the >75-age group, the Doctor’s note provided similar instructions as in the 50–75-years group.

#### Survey Sent to Participating Municipal Clinics

In order to help the public health care services to anticipate the potential workload that could be caused by the P5 Study, we contacted 143 clinical directors in municipal clinics in each of the 50 regions where the P5 Study participants originated from. The contact message summarising the upcoming study and providing examples of the Doctor’s notes was sent 2 weeks before releasing the first results to the portal. Moreover, 2 months after releasing the results we sent the same recipients a questionnaire related to workload that in reality manifested at the clinics by the P5 Study. The questionnaire consisted of two questions: “Did the P5 Study cause additional questions among the patients at your clinic?” and “Would you like to give additional feedback?”.

## Results

### The MyP5 Portal

We constructed a portal, a secure MyP5-webpage, for returning validated genetic risk and absolute disease risk information, including both common and rare genetic and traditional risk factors, to the study participants, and their physicians. The portal contains the following main sections: MyP5, The P5 study, Diseases, Polygenic risk score, Lifestyle, Heredity, and genes, When to see a physician?, and Contact information. Each section provides information on the specific topic. The MyP5 results section comprises of “My results”, “Questionnaires”, and “General results”. In addition, risk information was given to recruited P5 Study participants also in the form of a “Doctor’s note” to explain the results and to provide personal instructions on lifestyle management as well as possibility to print the “Doctor’s note” and show it to the treating physician.

### Participants Accessing the Portal

Of the population based study (FinHealth 2017) participants who received invitation to participate, 3,449 individuals (56%) participated in the P5 Study: 56% of the participants were women and 44% men. The majority of the participants were 55–75 years old. During the study, 26 participants dropped out for reasons related to health or not having access to a computer. Table 1 shows a comparison between FinHealth 2017 and P5 Study population characteristics.

**TABLE 1:** A comparison of participant characteristics between the FinHealth 2017 and P5 Studies. The information on FinHealth 2017 Study participants (age >30) was obtained from FinHeath 2017 Study report (Koponen et al., 2018).

	FinHealth 2017 study	P5 study	MyP5 portal participants	Never visited portal
N	8,217^a^	3,449	2,573	604
Men (%)	47	44	43	47
Age group (%)				
Age <30	9	7	8	2
Age 30–39	15	13	16	4
Age 40–49	15	15	17	7
Age 50–59	20	22	23	15
Age 60–69	23	26	24	31
Age 70–79	14	15	10	30
Age >80	6	3	1	10
Urban/Rural (%)	64/36	66/34	69/31	55/45
Educational level: comprehensive/intermediate/university (%)	21/35/44	15/33/52	11/31/58	33/39/28
BMI kg/m^2^ (mean)	27.5	27.2	26.9	28.4
Blood pressure >140/90 mmHg (%)	16	16	14	23
Cholesterol level >5 mmol/L (%)	55	55	56	56
Prevalent type 2 diabetes (%)	10	8	6	17
Prevalent coronary heart disease (%) (age >50)	6	3	3	5

a Total number of FInHealth Study individuals, not all have data on all variables.

The risk results were given to 3,177 participants who had genomic data available in a RCT setting in two Groups on November 19, 2019. Group 1 received both genetic risk and absolute 10-years risk of the diseases and Group 2 received a 10-years risk of diseases based on traditional risk factors only. Group 2 received the genetic risk and absolute 10-years risk on February 13, 2020. A 2 month interval between the Group 2 results was considered short enough so that the participants would still remember their reaction to their first results but long enough that they could have reflected the results, maybe also with their doctor, near relatives or friends. Seven per cent of original 3,449 participants did not eventually receive the 10-years disease risk estimates or PRS information. Reasons for this were related to either low yield in the DNA extraction process and/or quality control of Affymetrix genotyping array (separation in signal values was insufficient, scanned image was not produced, CEL file failed or damaged array). Of all the study participants (N = 3,177), 2,573 (81%) had visited the MyP5 portal by June 4, 2020. Some participants (N = 604) had not visited the portal at all. Table 1 compares the characteristics between the participants who visited the MyP5 portal with those who did not. During the first release of the results, 2,463 participants responded to the questionnaire before and 1,455 after receiving the results. In the second release of the results, in which only Group Two (N = 1,590) received new T2D genetic risk results, 1,186 responded to the questionnaire before and 462 after receiving the results. Participants were provided an opportunity to contact the research team via phone (4 h per week) or email in relation to any technical or medical questions. The total number of contacts for support was 114, of which 67 were by phone and 47 email. We categorized the types of contacting into “technical” (N = 103) and “medical” problems (N = 11). Half (N = 51) of the technical problems were related to the inability to access the portal, which were resolved by sending printed results on paper by letter to a total of 47 participants. The other half of the technical problems were about the inability to find the results or questionnaires on the portal. Medical problems were related to clarification of the participants’ results (especially SCV results) or the possibility to get results related to other diseases (e.g., Alzheimer’s or Parkinson’s disease).

### Ten-Year Disease Risk Estimates in the Portal

**FIGURE 1:**
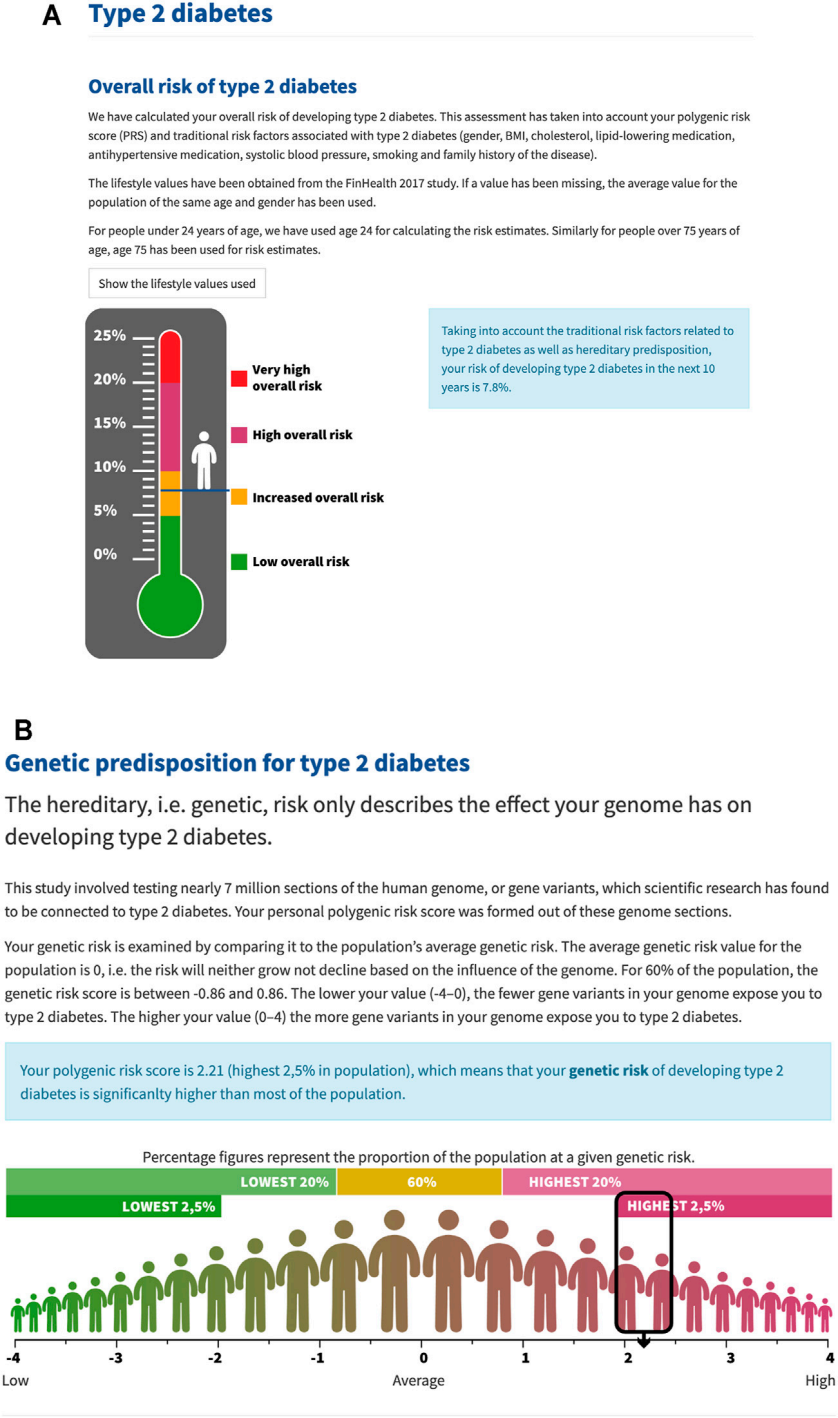
Layout of the portal. **(A)** The absolute (overall) risk was presented as a thermometer. The risk limits for T2D were 0–5% (low), 5–10% (increased), 10–20% (high), >20% (very high). **(B)** PRS was presented as a single value in relation to the whole population on a normal distribution curve.

We calculated a 10-years risk of T2D for each P5 Study participant using the point estimates and baseline hazard from the PRS model established in the FINRISK Study. The risk information was given for participants who did not have T2D and for those who did, but the results were clearly presented as the “risk in case the participant did not already have T2D”. For the majority of participants (60%), the risk was very low (0–5%). Figure 1 illustrates how the absolute risk and PRS alone was presented in the portal. In the portal, participants were able to see their traditional risk factors on which the risk was based on and a thermometer was used to present the absolute risk. Participant’s PRS value was shown on a normal distribution curve to illustrate participant´s own value in relation to the whole population.

### 10-Years T2D Risk Estimates at the Age of 60

**FIGURE 2:**
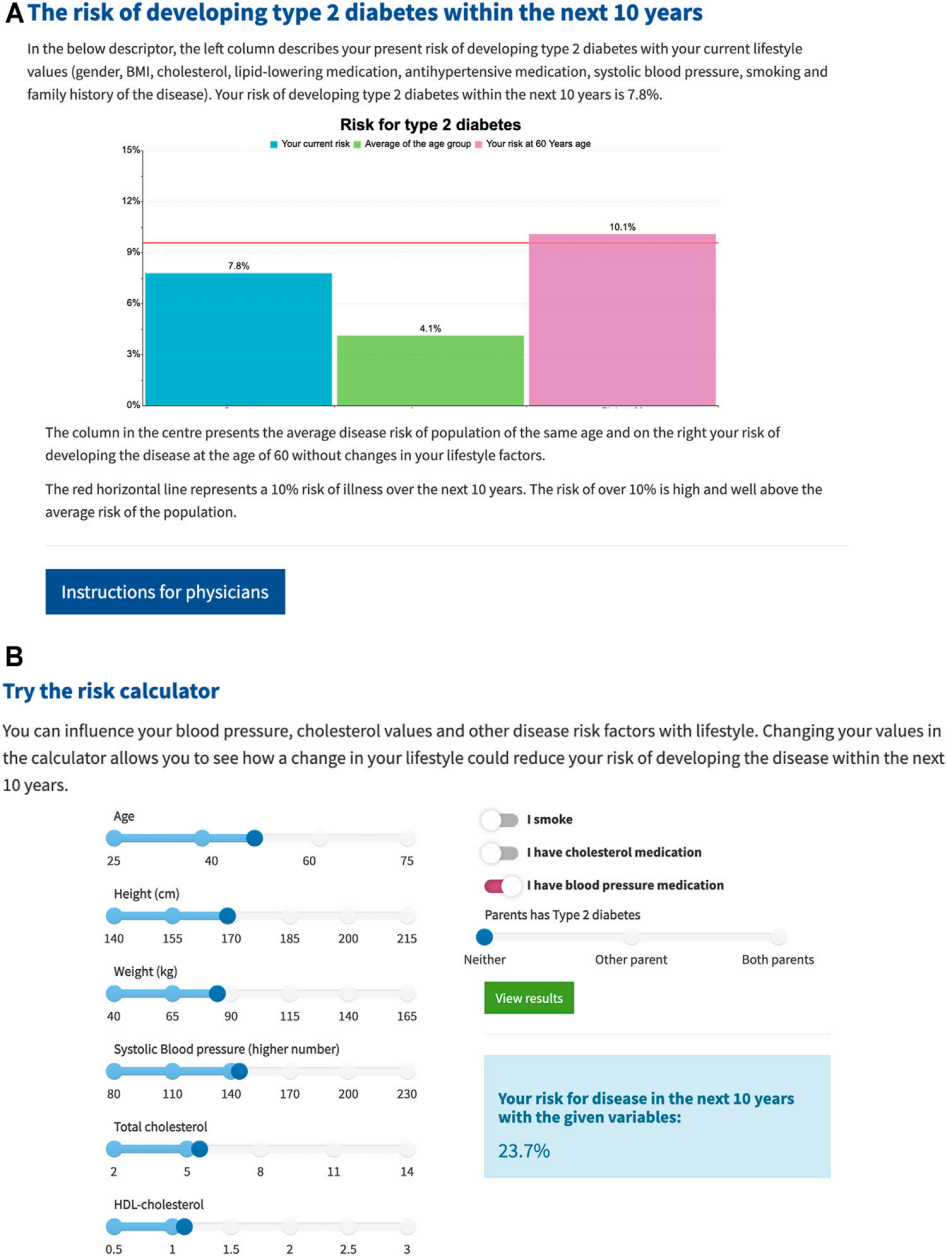
**(A)** The three columns from left to right present: participant’s absolute risk at current age, average absolute risk in similar age population and participant’s estimated 10-years risk at the age of 60. Red line at 10% presents the “high” risk threshold. **(B)** Participants were able to change their lifestyle factors in a risk calculator which could be seen as changes in the above risk columns.

Participants up to 50 years of age also received their absolute 10-years T2D risk at the age of 60 (Figure 2). The portal presented columns of current risk, average risk in a same age population and a 10-years risk estimation at the age of 60 assuming lifestyle factors would not change from current. All risk values were based on models including genetic and lifestyle factors. Participants were also able to try a risk calculator in which they could change their lifestyle factors to see how it affects the risk.

### Single Clinical Variants

All P5 Study participants received information on selected SCVs related to CHD or VTE. A total of 157 participants were heterozygote carriers of either of the two *F5* (N = 134) or *F2* (N = 23) mutations. They received a Doctor’s note that recommended mentioning the result to their physician. This note included information on the risk factors for developing deep vein thrombosis and how the risk can be reduced. In addition, it was stated that the variant is likely to appear in one’s family or relatives. Thus, it was advised that it would be useful to examine young women due to their higher VTE risk caused by possible hormonal contraception use and pregnancies, as well as those affected by a number of the risk factors mentioned in this letter.

In the case of either heterozygote (N = 1,047) or homozygote (N = 154) carriers of the pharmacogenetic variant in the *SLCO1B1* gene, a Doctor’s note recommended mentioning the result to a physician if the physician plans to start cholesterol medication or changes the existing treatment in the future. Participants were told not to give up any statin medication they were using at the time.

One participant who was homozygote for the *F5* variant and two participants who were heterozygotes for *LDLR* variant “FH Pogosta” were contacted in person by phone by a doctor to ensure that they had received and understood the result. They were also recommended to see a physician if they already hadn’t by that time. In addition, they were given instructions on how to confirm the carrier status result by giving a new blood sample via a health care-accredited laboratory (at no cost to the participant).

### Preliminary Feedback From Municipal Clinics and Participants

Two months after opening the portal, 143 clinical directors in municipal clinics in each of the 50 regions of the P5 Study participants were contacted with a questionnaire to gain information of the increased workload caused by the P5 Study. We received 44 (31%) replies of which 93% answered that the P5 Study did not increase workload at the clinics and the remaining 7% had received some questions from the P5 participants. Only one clinic answered that one participant had been directed to laboratory measurements.

The first feedback questionnaire for P5 participants included a question “Do you agree or disagree that the feedback you received on your results was difficult to understand?” A total of 1,354 participants answered to the question and 209 (16%) of them “did not agree or disagree”, 407 (30%) “fully disagreed”, 479 (35%) “somewhat disagreed”, 220 (16%) “somewhat agreed”, and 39 (3%) “fully agreed”.

## Discussion

Web portals for returning genetic results have been established by research studies as well as commercial companies (Tabor et al., 2017; 23andMe, 2021; Widén et al., 2020; Invitae, 2021; Mytoolboxgenomics, 2021). These portals offer information often referred to WGS (whole genome sequencing) data (McLaughlin et al., 2014; Tabor et al., 2017; Williams et al., 2018) or specific clinically relevant genetic variants (SNPs) (Haga et al., 2014; Solomon et al., 2020; Stefansdottir et al., 2020), and websites and apps in which one can calculate personal PRS values (Folkersen et al., 2020; MyGeneRank, 2021). These applications usually focus on genomic risk only and they do not provide personalized absolute risk estimates of future disease based on both genomic and clinical risk factors. There are widely used risk scores for CHD (Framingham Risk Score (D’Agostino et al., 2008), American College of Cardiology/American Heart Association 2013 risk score (ACC/AHA13)) (Stone et al., 2014)) and T2D [FINDRISC (Lindstrom and Tuomilehto, 2003)] that rely on clinical risk factors which have shown to be more efficient prediction tools to identify patients at risk of developing disease when genetic risk is added to them (Läll et al., 2017; Inouye et al., 2018). Specifically, even the applications utilizing only polygenic risk scores differ in predictive power, some integrating a genome-wide set of variants, and others a small set of selected variants either in population specific or more general reference population. The PRSs are constantly evolving, as new data from research becomes available and methods for calculating the scores advance. Furthermore, PRS studies performed in one population are not directly transferable to another (Reisberg et al., 2017; Duncan et al., 2019). Thus, it would be necessary to use up-to-date methods in PRS construction algorithms and design the protocols for interpretation of the results and their transfer to medical practice in the population the PRS will be implemented in. Also, return of genetic results should especially focus on the interpretation of the results to avoid potential misinterpretation of the genetic results and to avoid leaving the responsibility to the health care professionals in the daily clinical practice. Finally, as the results include sensitive health related personal data, the access security of web portals should be based on more than “username” and “password” authentication only.

To address these issues, we set up a secure web portal with content optimized for returning genetic information. To test the portal, we performed a study where we provided an opportunity for 3,449 volunteer participants to receive information on their genetic risk of T2D, CHD, and VTE. We tested a method that could be used to report PRS and SCV results to a large population, while preserving privacy with a secure “Suomi.fi e-Identification” identification system and keeping participants and, if they so wished, their health care providers informed on the patient-level clinical implications of the information. Through the web portal we presented participants information of the studied diseases, the concept of PRS, as well as the participant’s own estimated 10-years risk results based on both measured clinical and environmental risk factors and PRS in relation to the studied disease. Cardiometabolic diseases were selected for testing since they are common lifestyle diseases where preventive measures such as healthy lifestyles can be recommended to all risk classes and age groups.

Over half of the invited people took part in the P5 Study. The most actively participating individuals were those over 50 years old, suggesting that facilitating research and taking care of one’s health becomes more important with ageing. However, the future disease risk estimation would be especially useful for young adults, since early implementation of preventative measures most likely provides the greatest long-term benefits. For example, the prevalence of T2D has also increased in young adults over the past few years, thus highlighting the importance of preventative health care within adults under the age of 40 (Lascar et al., 2018). Here, genomic information could be used for early identification of those at increased risk prior to the symptoms of T2D, which would provide an opportunity to anticipate or even mitigate the risk for years or decades in advance. In this regard, the P5 Study participants also received a 10-years risk estimate at their hypothetical age of 60. It should be noted that mentioning the studied diseases in the invitation letter may introduce some selection bias to the P5 Study. It is however beyond the scope of this methods paper to analyze this further.

Interestingly, the participation rate of young adults in our study was lower than participation rates in older age groups. The portal was designed to work on a smartphone or tablet, thus the reason for not to participate is most likely not associated with an inability to access the portal. Possible reasons could be that the information to be received from the study was not interesting, relevant or preferred at that time, or concerns about data security. However, in the future, it will be important to engage young adults in possible future intervention efforts, since having knowledge of one’s future disease risk in advance could prevent or postpone the onset of disease.

The participants were able to contact our research group for medical or technical support. We received only 11 messages or phone calls related to clarification of the medical content of the results. Half of the technical problems (N = 51) were related to the lack of internet access and an inability to use the internet or the login identification system. We circumvented the issues by providing the results by letter for 47 participants, but if this kind of data-sharing method was taken as part of daily health care and more people would receive the results, a letter for a large number of people would require considerable resources. Furthermore, 604 participants did not visit the portal at all. A higher proportion of these participants seemed to have lower educational levels and belonged to the oldest age groups when compared to those who visited the portal. Moreover, since the proportion of participants who had already been diagnosed with T2D or CHD was larger among the participants who did not visit the portal, it could be that the information about one’s future disease risk was no longer relevant for some. Since education level and cardiometabolic risk factors are inversely correlated, it would be particularly important to encourage individuals in the lower education groups to participate in disease prevention efforts - both for the promotion of health equality and for public health as a whole (Dégano et al., 2017).

In addition to technical issues, returning genetic risk information to patients also involves ethical and social psychology considerations. In clinical genetics, an identified heritable genetic risk factor traditionally reveals information not only of the person studied but also of their family members. In the case of PRS, however, due to the polygenic nature of the score and the endless possibilities of the risk allele combinations, the actual relevance of PRS for family members remains an open question. From a psychological aspect, even if the PRS identifies the individuals at high risk for a given disease, getting to know one’s risk may not be sufficient to create a strong commitment to lifestyle changes but possible intervention or other support may be needed to achieve healthier lifestyle.

Effectively, the prevention of T2D or other common diseases affected both by lifestyle and genetic risk factors would be a patient-centric effort supported by health care professionals. To facilitate this, we created a “Doctor’s note” to explain the genetic risk results for physicians who are not necessarily experts in the field of genetics but might treat the participant. The Doctor’s note encouraged all those individuals with a 10-years T2D risk of over 10% to see a health care professional. The majority of the participants had a 0–5% risk of T2D, but about 20% of the participants had a risk of over 10%. Taking the Doctor’s note to the treating medical expert remained the choice of the participant. Our surveys to clinical directors in municipal clinics in each of the 50 regions where the P5 Study participants originated from revealed only few known contacts in the clinics by the participants. Monitoring how many of the high-risk individuals actually contacted a health care professional and were offered preventive interventions, and also how many of those who were not advised to contact health care also used health care services, will be carried out during a 5-year follow-up using national health registers and participant surveys. The information will be used to evaluate the costs and use of health care resources.

Our portal method was used not only to provide genetic risk results but also to perform a randomized controlled trial to study the effects on the participants’ health behavior. Previous studies have shown that receiving genetic risk information has varying effects on the recipient’s health behaviour (Hollands et al., 2016). The effect likely depends on the personal characteristics of individuals but also on how the information has been provided, as well as successfully tailored intervention efforts and support provided by health care professionals (Lindström et al., 2006; Lindström et al., 2013). Future efforts should be directed to studies on the impact of knowledge of the personal genetic risk on an individual’s health behaviour when supported with targeted interventions based on the PRS, and on comprehensive evaluation of the health implications from follow-up. These efforts should include study participants from varying age groups as age influences both disease risk and health behavior. Furthermore, the effect of genetic risk knowledge on behavior may vary depending on disease, thus it is important to compare effects across diseases and traits.

In our P5 study, questionnaires are used to observe how people react to receiving genomic information and how it affects their lifestyle choices thus enabling us to examine the possible psychosocial effects arising among the participants. In the future, our portal and the accompanying process of returning genetic risk results and estimates of future disease risk can be developed further to create online intervention programs for specific risk classes, invite individuals to face-to-face meetings, provide additional questionnaires, follow up the longitudinal health data in different risk categories, and even provide an opportunity to participants themselves to contact a health care professional through the portal.

In conclusion, we set up a secure web portal to conduct a proof of concept study on how PRS could be used as an additional biomarker of health—a personalised tool—for clinical work, especially in preventive health care. Our portal and its contents provide a platform that directly serves citizens and through them also health care professionals, while maintaining privacy. We demonstrated a way to report genetic risk information together with estimated future disease risk comprehensively so that not only citizens themselves could take advantage of it, but also it would provide guidelines for health care professionals who could easily translate the new information to patient care. Although in this study we focused especially on cardiometabolic diseases, our method could be easily expanded to report PRS related to other diseases or SCVs.

## Data Availability

Publicly available datasets were analyzed in this study. This data can be found here: https://thl.fi/en/web/thl-biobank/for-researchers.
