# Supplementary material for: A Web Portal for Communicating Polygenic Risk Score Results for Health Care Use—The P5 Study
Source: Front Genet. 2021 Oct 29;12:763159. doi: 10.3389/fgene.2021.763159 (PMC8585790; doi:10.3389/fgene.2021.763159)
Supplement: Supplementary file 1 [file DataSheet2.pdf]

Additional file 2

The National FINRISK Study for PRS evaluation

Characteristics of the FINRISK Study participants and analysis methods

To evaluate the association of the PRS with a future risk of T2D in the Finnish population, the population-based whole-genome genotyped National FINRISK Study was used (baseline years 1992, 1997, 2002, 2007, N=20,845) [1]. The FINRISK Studies are cross-sectional population surveys that were carried out every five years using an independent random sample to assess the risk factors of chronic diseases and health behaviour in the working-age population. Each study year comprises individuals of both sexes in 10-year age groups (25-34, 35-44, 45-54, 55-64, 65-74) within five (six in 2002) large study areas of Finland (Table 1). The cohorts have been followed up for >10 years by linking them to the national health registers. In the time-to-event analyses we used baseline information collected by questionnaires (smoking, family history of T2D), anthropometric measurements collected during the baseline health examinations (height, weight, blood pressure) and laboratory measurements (cholesterol, lipids). Data on incident events for T2D after the FINRISK Study baseline as well as medication (blood pressure, lipid-lowering medication) were obtained from national health care registers: the drug purchase register and the drug reimbursement register of the Social Insurance Institution of Finland (data from 1995-2017 and 2008-2017), the Population Register (1964–2017), Statistics Finland (1969–2017), and the Care Register for Health Care (1994–2017). Diagnoses were based on International Classification of Disease ICD9 and 10 (T2D: E11, 2502A, 2501A, 2503A, 2504A, 2505A, 2506A, 2507A, 2508A, 2500A) and drug reimbursement code E11 or drug purchase code A10B (T2D medication).

The FINRISK Studies have been genotyped with various Illumina (Illumina Inc., San Diego, CA, USA) genome-wide microchips. Genotyped data was quality-controlled to include individuals of Finnish origin by removing outliers in the principal component analysis and imputed to contain >17 M genomic variants using a Finnish high-quality whole-genome sequenced imputation reference panel SISu v3 (see FinnGen sample imputation in [2]).

Only incident cases (T2D: N=965) were used in the analysis, and prevalent cases were excluded. All statistical calculations were performed with R 3.6.0 (https://www.r-project.org). Survival curves and hazard ratios (HRs) with 95% confidence intervals (CI) were obtained from the Cox proportional hazards model. Since the aim was to create a method that could be implemented into real world health care, we built a model that would include variables normally available in standard clinical practice. For this reason, principal components depicting the genetic population structure, for example, were not used. The Cox regression analysis model for T2D was adjusted with traditional risk factors: gender, body mass index (BMI), total cholesterol, HDL, systolic blood pressure, blood pressure-lowering medication, lipid-lowering medication, self-reported family history of diabetes (0, 1 or 2 parents have diabetes) and smoking status (current smoker or not). The information on medication use at baseline was collected from FinHealth Study questionnaire data. Other covariates, the FINRISK Study year and study area were also used in the model. The proportionality of all variables was tested to ensure their constancy over time.

To select an optimal survival model, Akaike's Information Criterion (AIC) and Bayesian Information Criterion (BIC) techniques were used. To assess how well a given probability distribution and statistical regression model fits the data, thus the goodness of fit of the model, the Hosmer-Lemeshow test was used. Once an optimal model was determined, it was evaluated by the area under the curve (AUC) of a receiver operating a characteristic curve. Furthermore, to evaluate the added value of PRS in the model, the Net Reclassification Index (NRI) was used to assess the improved ability of a model containing the PRS to reclassify individuals into risk classes compared to a model containing the traditional risk factors only.

Table 1. Sample characteristics of the FINRISK cohort. Values are presented in mean ± standard deviation.

	Age 24-34 (N=4600)		Age 35-44 (N=4831)		Age 45-54 (N=4846)		Age 55-64 (N=4628)		Age 65-75 (N=1940)	
	Men (N=2003)	Women (N=2597)	Men (N=2165)	Women (N=2666)	Men (N=2251)	Women (N=2595)	Men (N=2168)	Women (N=2460)	Men (N=1040)	Women (N=900)
BMI (kg/m^2)	25.5±3.8	23.9±4.3	26.5±3.8	25.1±4.5	27.4±4.0	26.7±4.8	27.7±3.9	27.9±4.9	27.5±3.6	28.2±5.02
Total cholesterol (mmol/L)	5.1±1.0	4.9±0.9	5.6±1.0	5.2±0.9	5.9±1.1	5.6±1.0	5.7±1.1	6.0±1.0	5.5±1.0	5.9±1.04
HDL (mmol/L)	1.3±0.3	1.6±0.4	1.3±0.3	1.6±0.4	1.3±0.4	1.6±0.4	1.3±0.4	1.6±0.4	1.3±0.3	1.6±0.4
Systolic blood pressure (mmHg)	131.0±13.0	119.5±2.2	132.5±4.9	124.9±5.1	138.6±17.3	134.3±19.1	144.7±20.2	143.6±20.4	149.2±21.4	151.8± 21.2
Blood pressure medication (yes, %)	0.6	0.6	3.4	4.2	13.9	11.4	24.0	22.8	31.9	34.3
Lipid medication (yes, %)	0.1	0.0	1.8	0.3	4.7	1.9	12.6	8.0	18.8	19.9
Smoking (current smoker, %)	41.6	29.8	38.1	27.3	36.3	21.9	25.1	13.1	15.8	7.6
T2D cases within the 10-year time-frame from baseline (%)	1.0	1.0	2.9	1.5	7.5	4.4	9.3	6.5	8.5	9.4

Results of the analysis process of T2D

By using the Cox model, we examined the added value of PRS in the 10-year risk model by comparing a “basic” model including traditional factors only to a model with both traditional factors and PRS. AIC and BIC -values for the model containing the PRS were lower (14448.3 and 14526.2, respectively) compared to the basic model (14612.2 and 14685.3, respectively) suggesting that the model including the PRS performed better in estimating the 10-year risk. Since there were no statistically significant differences (p-value: 0.18, Hosmer-Lemeshow test) between the observed and expected cases, the PRS model was found to fit the FINRISK data. The AUC for the PRS model was 0.86 and for the basic model 0.85. We observed an association between PRS and T2D in the FINRISK cohort (HR: 1.5 per 1 sd increase, 95% CI: 1.43- 1.63, p-value: $<2*10^{-16}$). Since the effect of BMI on T2D incidence was non-proportional, an interaction with age was used to correct the proportionality. Detailed results of the Cox models are presented in the Table 2.

Table 2. Results of the time-to-event T2D survival model. Table shows the beta coefficients, hazard ratios (HRs), 95% confidence intervals (CI) and p-values of the most optimal Cox proportional-hazards model observed in the analyses. Cox model was adjusted with PRS and gender as well as traditional risk factors: BMI, total cholesterol, high-density lipoprotein (HDL), systolic blood pressure, blood pressure-lowering medication, lipid-medication, self-reported family history, smoking status and technical covariates: FINRISK survey collection year and study area.

	Beta coefficient	HR (95% CI)	p-value
T2D polygenic risk score	0.43	1.50 (1.43 - 1.63)	$3.04*10^{-38}$
Gender (male)	0.25	1.30 (1.11 - 1.48)	$5.95*10^{-4}$
BMI	0.24	1.30 (1.21 - 1.34)	$2.61*10^{-20}$
Cholesterol	0.16	1.20 (1.12 - 1.24)	$2.40*10^{-9}$
HDL	-0.91	0.40 (0.32 - 0.50)	$3.79*10^{-16}$
Systolic blood pressure	0.01	1.00 (1 .00- 1.01)	$2.79*10^{-6}$
Blood pressure medication (yes)	0.52	1.68 (1.45 - 1.94)	$3.12*10^{-12}$
Lipid medication (yes)	0.53	1.70 (1.41 - 2.06)	$4.70*10^{-8}$
Family history of T2D (one parent)<br>Family history of T2D (both parents)	0.37<br>0.47	1.45 (1.25 - 1.69)	$8.83*10^{-7}$<br>$3.34* 10^{-2}$
Smoking (yes)	0.24	1.60 (1.04 - 2.49)	$1.48*10^{-3}$
BMI:AGE interaction	-0.00	1.28 (1.10 - 1.48)	$1.09*10^{-4}$
AGE	-0.04	1.00 (1.00 - 1.00)	$5.55*10^{-2}$
FINRISK cohort -92<br>FINRISK cohort -97<br>(ref. FINRISK cohorts -02/-07)	-0.64<br>-0.29	0.96 (0.93 - 1.00)	$1.96*10^{-9}$<br>$1.30*10^{-4}$
Study areas in Finland (WEST)<br>(ref. EAST)	0.34	0.53 (0.43 - 0.65)	$5.74*10^{-7}$

We also looked at the C-index values of the T2D Cox model for all traditional risk factors individually (Fig. 1). We observed that smoking had the lowest C-index value (0.69; 95% CI:0.68-0.71) and BMI the highest (0.82; 95% CI:0.80-0.83). PRS alone had a C-index value of 0.74 (95% CI:0.72-0.75) and together with all traditional

risk factors the C-index was higher (0.85; 95% CI:0.84-0.87). The C-index for traditional risk factors only was 0.84 (95% CI:0.83-0.85).

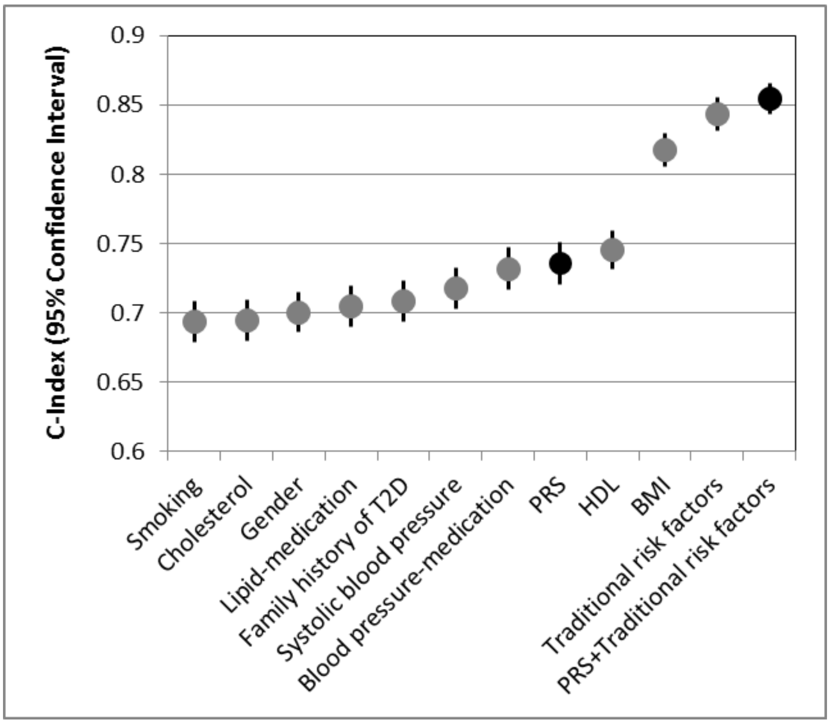

Fig 1. C-index values of the Cox model of T2D are presented for all traditional risk factors, PRS, basic model (traditional risk factors) and PRS model (PRS+ traditional risk factors) individually.

Cumulative differences in risk for T2D in five PRS categories

We compared T2D risk between five PRS groups: PRS <-1.5sd (N=1420), PRS between -1.5sd and -0.5sd (N=5224), PRS between -0.5sd and 0.5sd (N=8041), PRS between 0.5sd and 1.5sd (N=4786) and PRS >1.5sd (N=1374) (Fig.3). The lowest PRS group (PRS <-1.5 sd) was used as a reference. We detected that persons with PRS between >0.5sd and <1.5sd PRS had 2.9-fold increased risk for developing T2D within the next ten years (95% CI: 1.98-4.37, p-value: $8.42*10^{-8}$) compared to the reference, while persons with higher than 1.5sd PRS had over fourfold risk (HR: 4.5 per 1 sd, 95% CI: 2.98-6.83, p-value: $1.06*10^{-12}$). As shown by the Kaplan-Meier survival curve (Fig. 3) the cumulative risk differences can be clearly seen between the PRS groups already at the 2.5-year follow-up.

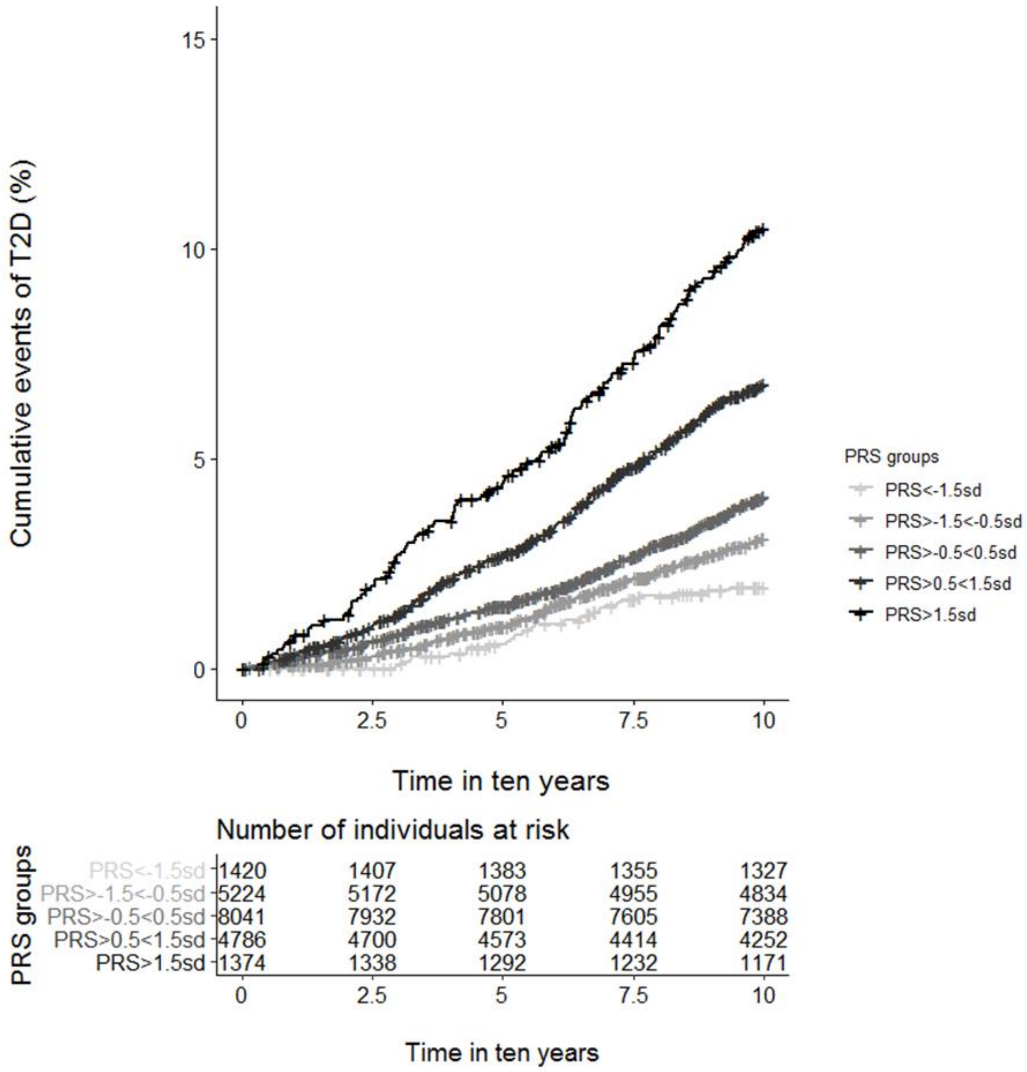

Fig 3. Kaplan-Meier survival curve of cumulative T2D events in a ten-year follow-up in five PRS groups. Lightest grey line presents the lowest PRS group and black line the highest PRS group.

PRS reclassifies individuals into new different risk classes compared to traditional risk factors

To see how employment of genetic risk information would potentially affect treatment or intervention strategies in healthcare, we compared risk category estimations from models with and without PRS using net reclassification improvement (NRI) analysis. First, 10-year T2D risk based on traditional risk factors only was calculated for all individuals: “Basic model”. Thereafter, individuals were divided into three risk classes when the 10-year risk for having T2D was either over 20%, between 10-20% or under 10%. According to the Finnish T2D treatment guidelines, we assume that in the high risk class (>20%) individuals would be already offered lifestyle intervention. Individuals in the lowest and intermediate risk categories would not receive any targeted care because of their lower risk estimation using traditional T2D risk factors.

After including the T2D PRS into the 10-year risk model we were able to categorize individuals into new, more specific, risk classes (Fig 4.). NRI for the whole sample was 0.07 (CI: 0.04-0.11, p-value: $5.3*10^{-6}$) and the result seemed to mainly reflect the improved reclassification of the T2D cases: for events the NRI was 0.07 (CI: 0.04-0.11, p-value: $3.4*10^{-6}$) and for nonevents -0.0 (CI: -0.00-0.00, p-value: 0.6). In the highest risk class, 78% of the individuals remained in the high risk category, but 21.4% moved into the intermediate risk class. Most of the individuals in the lowest risk class remained in this class and only a small proportion moved to the higher risk class. In contrast, in the intermediate risk class, 15% of the individuals moved to the higher risk class and 29% to the low-risk class.

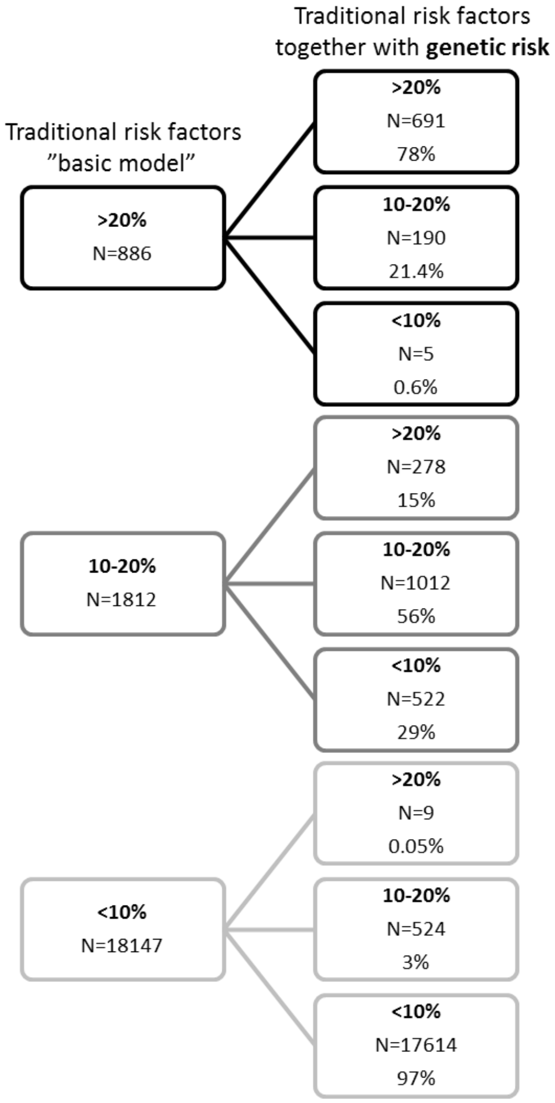

Fig. 4 Net reclassification improvement. FINRISK individuals were divided into three risk classes (<10% low, >10<20% intermediate, >20% high risk classes) based on their 10-year risk for developing T2D. On the left is the "basic" survival model containing only the traditional risk factors (BMI, total cholesterol, high-density lipoprotein (HDL), systolic blood pressure, blood pressure-lowering medication, lipid-medication, self-reported family history and smoking). Three boxes on the right present the new risk classes after reclassification, thus the number of individuals who are classified into new risk classes based on the survival model containing the PRS. Individuals are reclassifies especially in the intermediate risk class in which 15% of the individuals moved to the higher risk class and 29% to the low-risk class.

References

1. Borodulin K, Tolonen H, Jousilahti P, Jula A, Juolevi A, Koskinen S, Kuulasmaa K, Laatikainen T, Mannisto S, Peltonen M, Perola M, Puska P, Salomaa V, Sundvall J, Virtanen SM, Vartiainen E: **Cohort Profile: The National FINRISK Study.** Int J Epidemiol 2018, **47**(3):696-696i.

2. Tabassum R, Ramo JT, Ripatti P, Koskela JT, Kurki M, Karjalainen J, Palta P, Hassan S, Nunez-Fontarnau J, Kiiskinen TTJ, Soderlund S, Matikainen N, Gerl MJ, Surma MA, Klose C, Stitziel NO, Laivuori H, Havulinna AS, Service SK, Salomaa V, Pirinen M, FinnGen Project, Jauhiainen M, Daly MJ, Freimer NB, Palotie A, Taskinen MR, Simons K, Ripatti S: **Genetic architecture of human plasma lipidome and its link to cardiovascular disease.** Nat Commun 2019, **10**(1):4329-8.
