# Supplementary material for: A Web Portal for Communicating Polygenic Risk Score Results for Health Care Use—The P5 Study
Source: Front Genet. 2021 Oct 29;12:763159. doi: 10.3389/fgene.2021.763159 (PMC8585790; doi:10.3389/fgene.2021.763159)
Supplement: Supplementary file 2 [file DataSheet4.pdf]

This model for under-50-year-olds

Assessment of your risk of developing type 2 diabetes

You may present this information sheet to your physician

Dear NN,

Type 2 diabetes is caused by a metabolism dysfunction that decreases insulin production and reduces the effect of insulin. The disease gradually develops over several years, often without symptoms, and can be detected accidentally. As the disease progresses, the person's risk of developing a vascular disorder and other additional illnesses grows. Around 300,000 people are known to have type 2 diabetes in Finland. In addition to age, lifestyle and genetic factors influence the risk of developing the disease.

The information collected from you in the FinHealth study included your gender, BMI, cholesterol, lipid-lowering medication, antihypertensive medication, systolic blood pressure, smoking and family history of the disease, and used as the basis for calculating your risk of developing type 2 diabetes. We also used these values in this study. If one of these measurement results was not available for you, we used the average value of the population. We supplemented the risk assessment based on this information with a risk assessment based on genes. We examined nearly 7 million of your gene variants and used them to estimate your genetic risk of developing type 2 diabetes. The combined impact of the gene variants is described with the PRS value (polygenic risk score, or the combined impact of several different genetic factors). You can view your personal PRS, or polygenic risk score, in your personal results at the MyP5 website.

Different texts for 4 different risk groups:

<u>For the group: risk >20%</u>

Your MyP5 website includes our assessment of your likelihood for developing type 2 diabetes within the next ten years. The risk of type 2 diabetes grows as people age. As a result, we have also estimated your risk of developing diabetes based on your lifestyle and genes within the next ten years if you were currently 50 years old and had not yet been diagnosed with diabetes at that age. **Based on our estimate, your risk of developing type 2 diabetes at the age of 50–60 is over 20%, which is very high and clearly above the average risk level in the population.** In other words: out of one hundred people with a similar lifestyle and genes as you, more than 20 will probably develop diabetes at the age of 50–60. The risk assessment presumes that your lifestyle remains as it is currently. This assessment has not taken into account very rare gene variants affecting inherited forms of diabetes.

We recommend that you show this message to a physician or public health nurse at a health centre or in occupational health care, for instance. A risk assessment this high indicates that you are at a risk of developing the disease at a much younger age than average. As a result, it would be highly important for the health care services to evaluate on a case-by-case basis what sort of guidance and support you need in

implementing lifestyle changes and when there will be need to monitor your blood values to detect the onset of diabetes.

Your genes stay the same throughout your entire life. However, a healthy lifestyle can highly significantly reduce your risk of developing a disease or postpone the onset of a disease by several years. It is particularly important to engage in physical activity, eat healthily and aim at maintaining a normal weight. Even a minor weight loss will reduce the risk. It is also important not to smoke. Good instructions are available at the Finnish Diabetes Association website at www.diabetes.fi.

For the group: risk >10% <20%

Your MyP5 website includes our assessment of your likelihood for developing type 2 diabetes within the next ten years. The risk of type 2 diabetes grows as people age. As a result, we have also estimated your risk of developing diabetes based on your lifestyle and genes within the next ten years if you were currently 50 years old and had not yet been diagnosed with diabetes at that age. **Based on our estimate, your risk of developing type 2 diabetes at the age of 50–60 is over 10–20%, which is high and above the average risk level in the population.** In other words: out of one hundred people with a similar lifestyle and genes as you, around 10–20 will probably develop diabetes at the age of 50–60. The risk assessment presumes that your lifestyle remains as it is currently. This assessment has not taken into account very rare gene variants affecting inherited forms of diabetes.

We recommend that you show this message to a physician or public health nurse at a health centre or in occupational health care, for instance. A risk assessment this high indicates that you may be at a risk of developing the risk before the age of 50–60. As a result, it would be good for the health care services to evaluate on a case-by-case basis what sort of guidance and support you need in implementing lifestyle changes and when there will be need to monitor your blood values to detect the onset of diabetes.

Your genes stay the same throughout your entire life. However, a healthy lifestyle can highly significantly reduce your risk of developing a disease or postpone the onset of a disease by several years. It is particularly important to engage in physical activity, eat healthily and aim at maintaining a normal weight. Even a minor weight loss will reduce the risk. It is also important not to smoke. Good instructions are available at the Finnish Diabetes Association website at www.diabetes.fi.

For the group: risk >5% <10%

Your MyP5 website includes our assessment of your likelihood for developing type 2 diabetes within the next ten years. The risk of type 2 diabetes grows as people age. As a result, we have also estimated your risk of developing diabetes based on your lifestyle and genes within the next ten years if you were currently 50 years old and had not yet been diagnosed with diabetes at that age. **Based on our estimate, your risk of developing type 2 diabetes at the age of 50–60 is 5–10%. This is a slightly elevated result and above the average population risk.** In other words: out of one hundred people with a similar lifestyle and genes as you, 5–10 will probably develop diabetes at the age of 50–60. The risk assessment presumes that your lifestyle remains as it is currently. This assessment has not taken into account very rare gene variants affecting inherited forms of diabetes.

Your genes stay the same throughout your entire life. However, a healthy lifestyle can highly significantly reduce your risk of developing a disease or postpone the onset of a disease by several years. It is particularly important to engage in physical activity, eat healthily and aim at maintaining a normal weight. Even a minor weight loss will reduce the risk. It is also important not to smoke.

You can show this message to a physician or public health nurse at a health centre or in occupational health care, for instance, to receive guidance and support for implementing lifestyle changes. Good instructions are available at the Finnish Diabetes Association website at www.diabetes.fi.

For the group: risk <5%

Your MyP5 website includes our assessment of your likelihood for developing type 2 diabetes within the next ten years. The risk of type 2 diabetes grows as people age. As a result, we have also estimated your risk of developing diabetes based on your lifestyle and genes within the next ten years if you were currently 50 years old and had not yet been diagnosed with diabetes at that age. **Based on our estimate, your risk of developing type 2 diabetes at the age of 50–60 is less than 5%. This result is equal to the average population rates.** In other words: out of one hundred people with a similar lifestyle and genes as you, less than 5 will probably develop diabetes at the age of 50–60. The risk assessment presumes that your lifestyle remains as it is currently. This assessment has not taken into account very rare gene variants affecting inherited forms of diabetes.

Your genes stay the same throughout your entire life. However, a healthy lifestyle can highly significantly reduce your risk of developing a disease or postpone the onset of a disease by several years. It is particularly important to engage in physical activity, eat healthily and aim at maintaining a normal weight. It is also important not to smoke. Good instructions are available at the Finnish Diabetes Association website at www.diabetes.fi.

This model for 50-75-year-olds

Assessment of your risk of developing type 2 diabetes

You may present this information sheet to your physician.

Dear NN,

Type 2 diabetes is caused by a metabolism dysfunction that decreases insulin production and reduces the effect of insulin. The disease gradually develops over several years, often without symptoms, and can be detected accidentally. As the disease progresses, the person's risk of developing a vascular disorder and other additional illnesses grows. Around 300,000 people are known to have type 2 diabetes in Finland. In addition to age, lifestyle and genetic factors influence the risk of developing the disease.

The information collected from you in the FinHealth study included your gender, BMI, cholesterol, lipid-lowering medication, antihypertensive medication, systolic blood pressure, smoking and family history of the disease, and used as the basis for calculating your risk of developing type 2 diabetes. We also used these values in this study. If one of these measurement results was not available for you, we used the average value of the population. We supplemented the risk assessment based on this information with a risk assessment based on genes. We examined nearly 7 million of your gene variants and used them to estimate your genetic risk of developing type 2 diabetes. The combined impact of the gene variants is described with the PRS value (polygenic risk score, or the combined impact of several different genetic factors). You can view your personal PRS, or polygenic risk score, in your personal results at the MyP5 website.

Different texts for 4 different risk groups:

<u>For the group: risk >20%</u>

The risk of type 2 diabetes grows as people age. Below, we evaluate your risk of developing type 2 diabetes within the next ten years if you have not yet been diagnosed with diabetes. **Based on our estimate, your risk of developing type 2 diabetes within the next ten years is over 20%, which is very high and clearly above the average risk level in the population.** In other words: out of one hundred people with a similar lifestyle and genes as you, more than 20 will probably develop type 2 diabetes within the next ten years. The risk assessment presumes that your lifestyle remains as it is currently. This assessment has not taken into account very rare gene variants affecting inherited forms of diabetes.

We recommend that you show this message to a physician or public health nurse at a health centre or in occupational health care, for instance. We think it would be highly important for the health care services to evaluate on a case-by-case basis what sort of guidance and support you need in implementing lifestyle changes and when there will be need to monitor your blood values to detect the onset of diabetes.

Your genes stay the same throughout your entire life. However, a healthy lifestyle can highly significantly reduce your risk of developing a disease or postpone the onset of a disease by several years. It is particularly important to engage in physical activity, eat healthily and aim at maintaining a normal weight.

Even a minor weight loss will reduce the risk. It is also important not to smoke. Good instructions are available at the Finnish Diabetes Association website at www.diabetes.fi.

For the group: risk >10% <20%

The risk of type 2 diabetes grows as people age. Below, we evaluate your risk of developing type 2 diabetes within the next ten years if you have not yet been diagnosed with diabetes. **Based on our estimate, your risk of developing type 2 diabetes within the next ten years is 10–20%, which is high compared to the average risk level in the population.** In other words: out of one hundred people with a similar lifestyle and genes as you, 10–20 will probably develop type 2 diabetes within the next ten years. The risk assessment presumes that your lifestyle remains as it is currently. This assessment has not taken into account very rare gene variants affecting inherited forms of diabetes.

We recommend that you show this message to a physician or public health nurse at a health centre or in occupational health care, for instance. We think it would be good for the health care services to evaluate on a case-by-case basis what sort of guidance and support you need in implementing lifestyle changes and when there will be need to monitor your blood values to detect the onset of diabetes.

Your genes stay the same throughout your entire life. However, a healthy lifestyle can highly significantly reduce your risk of developing a disease or postpone the onset of a disease by several years. It is particularly important to engage in physical activity, eat healthily and aim at maintaining a normal weight. Even a minor weight loss will reduce the risk. It is also important not to smoke. Good instructions are available at the Finnish Diabetes Association website at www.diabetes.fi.

For the group: risk >5% <10%

The risk of type 2 diabetes grows as people age. Below, we evaluate your risk of developing type 2 diabetes within the next ten years if you have not yet been diagnosed with diabetes. **Based on our estimate, your risk of developing type 2 diabetes within the next ten years is 5–10 %. While this is not particularly high compared to the average population risk, it is nevertheless worth considering.** In other words: out of one hundred people with a similar lifestyle and genes as you, 5–10 will probably develop diabetes within the next ten years. The risk assessment presumes that your lifestyle remains as it is currently. This assessment has not taken into account very rare gene variants affecting inherited forms of diabetes.

Your genes stay the same throughout your entire life. However, a healthy lifestyle can highly significantly reduce your risk of developing a disease or postpone the onset of a disease by several years. It is particularly important to engage in physical activity, eat healthily and aim at maintaining a normal weight. Even a minor weight loss will reduce the risk. It is also important not to smoke.

You can show this message to a physician or public health nurse at a health centre or in occupational health care, for instance, to receive guidance and support for implementing lifestyle changes. Good instructions are available at the Finnish Diabetes Association website at www.diabetes.fi.

For the group: risk <5%

The risk of type 2 diabetes grows as people age. Below, we evaluate your risk of developing type 2 diabetes within the next ten years if you have not yet been diagnosed with diabetes. **Based on our estimate, your risk of developing type 2 diabetes within the next ten years is less than 5%. In your age group, this is a fairly low risk compared to the average population risk.** In other words: out of one hundred people with a similar lifestyle and genes as you, fewer than 5 will probably develop type 2 diabetes within the next ten years. The risk assessment presumes that your lifestyle remains as it is currently. This assessment has not taken into account very rare gene variants affecting inherited forms of diabetes.

Your genes stay the same throughout your entire life. However, **a healthy lifestyle can further reduce your risk of developing a disease or postpone the onset of a disease by several years.** It is particularly important to engage in physical activity, eat healthily and aim at maintaining a normal weight. Even a minor weight loss will reduce the risk. It is also important not to smoke. Good instructions are available at the Finnish Diabetes Association website at www.diabetes.fi.

This model for over-75-year-olds.

Assessment of your risk of developing type 2 diabetes

You may present this information sheet to your physician

Dear NN,

Type 2 diabetes is caused by a metabolism dysfunction that decreases insulin production and reduces the effect of insulin. The disease gradually develops over several years, often without symptoms, and can be detected accidentally. As the disease progresses, the person's risk of developing a vascular disorder and other additional illnesses grows. Around 300,000 people are known to have type 2 diabetes in Finland. In addition to age, lifestyle and genetic factors influence the risk of developing the disease.

The information collected from you in the FinHealth study included your gender, BMI, cholesterol, lipid-lowering medication, antihypertensive medication, systolic blood pressure, smoking and family history of the disease, and used as the basis for calculating your risk of developing type 2 diabetes. We also used these values in this study. If one of these measurement results was not available for you, we used the average value of the population. We supplemented the risk assessment based on this information with a risk assessment based on genes. We examined nearly 7 million of your gene variants and used them to estimate your genetic risk of developing type 2 diabetes. The combined impact of the gene variants is described with the PRS value (polygenic risk score, or the combined impact of several different genetic factors). You can view your personal PRS, or polygenic risk score, in your personal results at the MyP5 website.

Different texts for 4 different risk groups:

<u>For the group: risk >20%</u>

The risk of type 2 diabetes grows as people age. **According to our results, at the age of 75, your risk of developing diabetes within the next ten years was over 20%.** In other words: out of one hundred people aged 75 with a similar lifestyle and genes as you, more than 20 will probably develop diabetes within a few coming years. The risk assessment presumes that the persons have not yet been diagnosed with diabetes and their lifestyle remains as it is currently. **This risk is high, and it can also be considered indicative at your current age even though it is not possible to calculate a clear risk assessment to research participants over 75 years of age based on our research data.** This assessment has not taken into account very rare gene variants affecting inherited forms of diabetes.

We recommend that you show this message to a physician or public health nurse at a health centre, for instance. We think it would be highly important for the health care services to evaluate on a case-by-case basis what sort of guidance and support you need in implementing lifestyle changes and when there will be need to monitor your blood values to detect the onset of diabetes.

Your genes stay the same throughout your entire life. However, a healthy lifestyle can highly significantly reduce your risk of developing a disease or postpone the onset of a disease by several years. It is

particularly important to engage in physical activity, eat healthily and aim at maintaining a normal weight. Even a minor weight loss will reduce the risk. It is also important not to smoke. Good instructions are available at the Finnish Diabetes Association website at www.diabetes.fi.

For the group: risk >10% <20%

The risk of type 2 diabetes grows as people age. **According to our results, at the age of 75, your risk of developing diabetes within the next ten years was over 10–20%.** In other words: out of one hundred people aged 75 with a similar lifestyle and genes as you, 10–20 will probably develop diabetes within a few coming years. The risk assessment presumes that the persons have not yet been diagnosed with diabetes and their lifestyle remains as it is currently. **This risk is fairly high, and it can also be considered indicative at your current age even though it is not possible to calculate a clear risk assessment to research participants over 75 years of age based on our research data.** This assessment has not taken into account very rare gene variants affecting inherited forms of diabetes.

We recommend that you show this message to a physician or public health nurse at a health centre, for instance. We think it would be good for the health care services to evaluate on a case-by-case basis what sort of guidance and support you need in implementing lifestyle changes and when there will be need to monitor your blood values to detect the onset of diabetes.

Your genes stay the same throughout your entire life. However, a healthy lifestyle can highly significantly reduce your risk of developing a disease or postpone the onset of a disease by several years. It is particularly important to engage in physical activity, eat healthily and aim at maintaining a normal weight. Even a minor weight loss will reduce the risk. It is also important not to smoke. Good instructions are available at the Finnish Diabetes Association website at www.diabetes.fi.

For the group: risk >5% <10%

The risk of type 2 diabetes grows as people age. **According to our results, at the age of 75, your risk of developing diabetes within the next ten years was over 5-10 %.** In other words: out of one hundred people aged 75 with similar lifestyle and genes as you, 5-10 will probably develop diabetes within a few coming years. The risk assessment presumes that the persons have not yet been diagnosed with diabetes and their lifestyle remains as it is currently. **While this risk is not high compared to the average population risk, it is nevertheless worth considering. It can also be considered indicative at your current age even though it is not possible to calculate a clear risk assessment to research participants over 75 years of age based on our research data.** This assessment has not taken into account very rare gene variants affecting inherited forms of diabetes.

Your genes stay the same throughout your entire life. However, a healthy lifestyle can highly significantly reduce your risk of developing a disease or postpone the onset of a disease by several years. It is particularly important to engage in physical activity, eat healthily and aim at maintaining a normal weight. Even a minor weight loss will reduce the risk. It is also important not to smoke.

You can show this message to a physician or public health nurse at a health centre, for instance, to receive guidance and support for implementing lifestyle changes. Good instructions are available at the Finnish Diabetes Association website at www.diabetes.fi.

For the group: risk <5%

The risk of type 2 diabetes grows as people age. **According to our results, at the age of 75, your risk of developing diabetes within the next ten years was less than 5%.** In other words: out of one hundred people aged 75 with a similar lifestyle and genes as you, less than 5 will probably develop diabetes within a few coming years. The risk assessment presumes that the persons do not yet have diabetes and their lifestyle remains as it is currently. **This risk is fairly low in the age group of 75-year-olds, and it can also be considered indicative at your current age even though it is not possible to calculate a clear risk assessment to research participants over 75 years of age based on our research data.** This assessment has not taken into account very rare gene variants affecting inherited forms of diabetes.

Your genes stay the same throughout your entire life. However, **a healthy lifestyle can further reduce your risk of developing a disease or postpone the onset of a disease by several years.** It is particularly important to engage in physical activity, eat healthily and aim at maintaining a normal weight. Even a minor weight loss will reduce the risk. It is also important not to smoke. Good instructions are available at the Finnish Diabetes Association website at www.diabetes.fi.
