# Supplementary material for: A Web Portal for Communicating Polygenic Risk Score Results for Health Care Use—The P5 Study
Source: Front Genet. 2021 Oct 29;12:763159. doi: 10.3389/fgene.2021.763159 (PMC8585790; doi:10.3389/fgene.2021.763159)
Supplement: Supplementary file 3 [file DataSheet3.pdf]

QUESTIONNAIRE 1 BEFORE GIVING ANSWERS

Before redirecting you to your personal information, we would like to ask you a few questions. After you have provided the information, we will ask you more questions, which aim at determining how comprehensibly the health information has been presented and how useful our research participants have found it.

1. How do you perceive your personal risk of developing a cardiovascular disease?

1 very high
2 high
3 moderate
4 low
5 very low
6 I have been previously diagnosed with a cardiovascular disease

2. How much do you think you can influence whether or not you will develop a cardiovascular disease?

**Not at all**						**Very much**
1	2	3	4	5	6	7

3. How do you perceive your personal risk of developing type 2 diabetes?

1 very high
2 high
3 moderate
4 low
5 very low
6 I have been previously diagnosed with diabetes

4. How much do you think you can influence whether or not you will develop type 2 diabetes?

**Not at all**						**Very much**
1	2	3	4	5	6	7

5. How do you perceive your personal risk of developing a deep vein thrombosis in your calf or some other body part?

1 very high
2 high
3 moderate

4 low
5 very low
6 I have been previously diagnosed with a deep vein thrombosis in my calf or some other body part

6. How much do you think you can influence whether or not you will develop a deep vein thrombosis in your calf or some other body part?

**Not at all**						**Very much**
1	2	3	4	5	6	7

7. How concerned are you about your risk of developing these diseases

	**Not at all worried**						**Deeply worried**
Cardiovascular disease	1	2	3	4	5	6	7
Type 2 diabetes	1	2	3	4	5	6	7
Deep vein thrombosis in my calf or some other body part	1	2	3	4	5	6	7

8. Generally speaking, how much do you like taking risks?

Not at all	0	1	2	3	4	5	6	7	8	9	10	Very much

9. How willing are you to give up something beneficial to you to get greater benefits in the future?

Completely unwilling 0 1 2 3 4 5 6 7 8 9 10 Highly willing

QUESTIONNAIRE 1. AFTER RESPONDING

1. Express your opinion on the following statements. Do you agree or disagree that the feedback you received on your results was:

Select the appropriate response:

	Fully disagree	Somewhat disagree	Neither agree disagree	Somewhat disagree	Fully agree
Interesting	1	2	3	4	5
Difficult to understand	1	2	3	4	5
Useful	1	2	3	4	5
Worrying	1	2	3	4	5
Reliable information	1	2	3	4	5
New information about your health	1	2	3	4	5
Changed your view about your health	1	2	3	4	5
Encouraged you to cons changing your lifestyle	1	2	3	4	5

2. How do you perceive your personal risk of developing a cardiovascular disease?

1 very high
2 high
3 moderate
4 low
5 very low
6 I have been previously diagnosed with a cardiovascular disease

3. How much do you think you can influence whether or not you will develop a cardiovascular disease?

**Not at all**						**Very much**
1	2	3	4	5	6	7

4. How do you perceive your personal risk of developing a type 2 diabetes?

1 very high
2 high
3 moderate
4 low
5 very low

6 I have been previously diagnosed with diabetes

5. How much do you think you can influence whether or not you will develop type 2 diabetes?

Not at all						Very much
1	2	3	4	5	6	7

6. How do you perceive your personal risk of developing a thrombosis in your calf or some other body part?

1 very high
2 high
3 moderate
4 low
5 very low
6 I have been previously diagnosed with a deep vein thrombosis in my calf or some other body part

7. How much do you think you can influence whether or not you will develop a deep vein thrombosis in your calf or some other body part?

Not at all						Very much
1	2	3	4	5	6	7

8. How worried are you about your risk based on "traditional" measurement results (incl. cholesterol)?

	Not at all worried						Deeply worried
Cardiovascular disease	1	2	3	4	5	6	7
Type 2 diabetes	1	2	3	4	5	6	7
Deep vein thrombosis in my calf or some other body part	1	2	3	4	5	6	7

10. How worried are you about your risk based on your genome?

	**Not at all worried**						**Deeply worried**
Cardiovascular disease	1	2	3	4	5	6	7
Type 2 diabetes	1	2	3	4	5	6	7
Deep vein thrombosis in my calf or some other body part	1	2	3	4	5	6	7

11. I am planning to increase my physical activity within the next four weeks

1. Very unlikely
2. Unlikely
3. Neither likely nor unlikely
4. Likely
5. Very likely

12. I intend to eat more fruit and vegetables within the next four weeks

1. Very unlikely
2. Unlikely
3. Neither likely nor unlikely
4. Likely
5. Very likely

13. Have you sought a medical examination or treatment (with a physician, a public health nurse or a nurse) based on your previous FinHealth 2017 study results (health examination or health profile letter)?

1. No, I have not
2. I might do that
3. Yes, I have

14. Do you intend to seek a medical examination or treatment (with a physician, a public health nurse or a nurse) based on the feedback you received from the P5 study?

1. No
2. Maybe
3. Yes

15. The following scale consists of words that describe an emotion or mood. Select the number that best describes how strongly you are feeling each emotion right now.

	**Very little or not at all**	**A little**	**Somewhat**	**Quite a lot**	**Very much**
1. I feel shocked about my test result.	1	2	3	4	5
2. I feel sad about my test result.	1	2	3	4	5
3. I feel nervous or anxious about my test result.	1	2	3	4	5
4. I feel guilty about my test result.	1	2	3	4	5
5. I feel relieved about my test result.	1	2	3	4	5
6. I feel happy about my test result.	1	2	3	4	5
7. I feel a loss of control because of my test result.	1	2	3	4	5
8. I have problems enjoying life because of my test result.	1	2	3	4	5

13. I can effectively prevent developing type 2 diabetes by engaging in physical activity.

1. Fully disagree
2. Somewhat disagree
3. Neither agree nor disagree
4. Somewhat agree
5. Fully agree

14. I can effectively prevent developing a cardiovascular disease by engaging in physical activity.

1. Fully disagree

2. Somewhat disagree
3. Neither agree nor disagree
4. Somewhat agree
5. Fully agree

15. I can effectively prevent developing type 2 diabetes by eating a lot of fruit and vegetables.

1. Fully disagree
2. Somewhat disagree
3. Neither agree nor disagree
4. Somewhat agree
5. Fully agree

16. I can effectively prevent developing a cardiovascular disease by eating a lot of fruit and vegetables.

1. Fully disagree
2. Somewhat disagree
3. Neither agree nor disagree
4. Somewhat agree
5. Fully agree

17. How often have you eaten vegetables (not including potatoes) within the previous 7 days as such, grated or in a fresh salad?

1. Not at all
2. 1 to 2
3. 3 to 5 days
4. on 6 to 7 days
5. Several times a day

18. How often have you eaten fruit or berries within the past 7 days?

1. Not at all
2. 1 to 2
3. 3 to 5 days
4. on 6 to 7 days
5. Several times a day

19. Which of the following statements describe your food selections? It is important to me

	**Not at all important**	**Not very important**	**Cannot say**	**Quite important**	**Very important**
1. that my diet includes a lot of meat products	1	2	3	4	5
2. that my food contains little additives	1	2	3	4	5
3. to select foods that contain little fat	1	2	3	4	5
4. to favour high-fibre foods	1	2	3	4	5
5. to avoid food with high salt content	1	2	3	4	5
6. to follow a low carbohydrate diet	1	2	3	4	5

20. Do you smoke currently (cigarettes, cigars or pipe)?

1. Yes, daily
2. Yes, occasionally
3. Not at all

21. When was the last time you smoked? If you smoke constantly, please select the option "yesterday or today".

1. Yesterday or today
2. 2 days to 1 year ago
3. 1 month to 6 months ago
4. 6 months to one year ago
5. 1 to 5 years ago
6. 6 to 10 years ago
7. More than 10 years ago
8. Never

22. If you still smoke, how willing would you be to permanently give up smoking?

Not at all willing	1	2	3	4	5	6	7	8	9	10	Highly willing

23. How often do you drink alcoholic beverages?

1. Never
2. Once a month or less frequently
3. 2–4 times a month
4. 2–3 times a week
5. 4 times or more a week

24. How many drinks containing alcohol have you drunk on a typical day when you have been drinking? One portion is:
0.33 l medium-strength beer, cider or long drink, max 4.7%
12 cl of regular wine, 10–15%
8 cl of fortified wine, 16–22%
4 cl of fortified wine, 35–40%

1. 1–2 servings
2. 3–4 servings
3. 5–6 servings
4. 7–9 servings
5. 10 or more servings

Physical activity

25. Overall, how physically active are you during the week at work, on your ways to and from work, and during your leisure time? Think about the previous three weeks. Take into account all regular weekly physical activity which lasts at least 10 minutes per session. You can write your answer on several lines if necessary.

1. Hardly any regular activity each week
2. Calm and low-intensity physical activity (= does not make you sweat or get out of breath, e.g. slow walking) _____ days per week, in total _____ hours and _____ minutes per week
3. Brisk and vigorous physical activity (= makes you sweat a bit and/or get slightly out of breath, e.g. brisk walking) _____ days per week, in total _____ hours and _____ minutes per week

4. High-intensity aerobic physical activity (= makes you sweat a lot and/or get out of breath, e.g. jogging or running) _____ days per week, in total _____ hours and _____ minutes per week

26. On how many days during an ordinary week do you engage in exercise that maintains muscles or enhances muscle tone? For instance, exercising at a gym, home exercises, fitness classes, ball games and racked sports or physically straining household chores. Think about the past 12 months. If you do not engage in any exercise, please write '0'.

_____days per week

Weight and weight loss

27. What is your current weight? ________ kg

28. Have you ever seriously tried losing weight? If yes, how many times?

1. I have never tried losing weight
2. 1–2 times
3. 3–5 times
4. 6 times or more

29. What was your best weight loss result when you were attempting to lose weight?

5. Less than 2 kg
6. 2–5 kg
7. 6–10 kg
8. More than 10 kg

30. Are you currently trying to lose weight?

1. No
2. Yes

31. Below are listed some statements regarding emotions and thoughts. For each statement, select the alternative that best describes your experiences within the past 2 weeks.

	**Not at all**	**Rarely**	**Now and then**	**Often**	**All the time**
1. I have felt hopeful about the future	1	2	3	4	5
2. I have felt useful	1	2	3	4	5
3. I have felt relaxed	1	2	3	4	5
4. I have been interested in other people.	1	2	3	4	5
5. I have had a lot of energy	1	2	3	4	5
6. I have dealt with problems well	1	2	3	4	5
7. I have been thinking clearly	1	2	3	4	5
8. I have been feeling good about myself	1	2	3	4	5
9. I have felt closeness with other people	1	2	3	4	5
10. I have been feeling confident.	1	2	3	4	5
11. I have managed to make my own decisions on things	1	2	3	4	5
12. I have been feeling loved	1	2	3	4	5
13. I have been interested in new things					
14. I have been feeling cheerful					

32. Finally, you can use the below space to provide feedback about the information you were given on your health.
What should we pay attention to when giving people feedback about their health?
