# Supplementary material for: A Web Portal for Communicating Polygenic Risk Score Results for Health Care Use—The P5 Study
Source: Front Genet. 2021 Oct 29;12:763159. doi: 10.3389/fgene.2021.763159 (PMC8585790; doi:10.3389/fgene.2021.763159)
Supplement: Supplementary file 4 [file DataSheet1.pdf]

Workflow

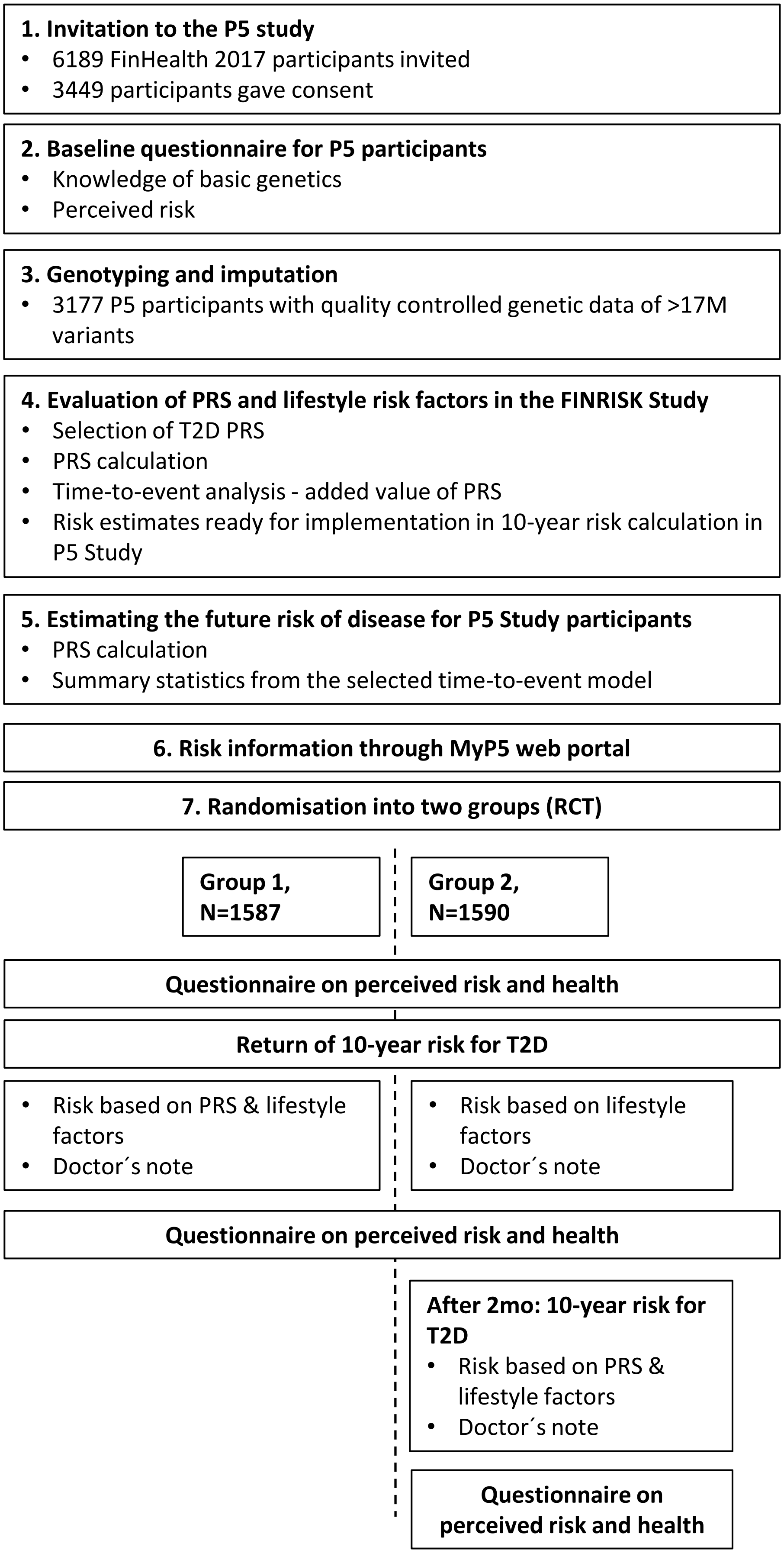
1. Invitation to the P5 study
6189 FinHealth 2017 participants invited
3449 participants gave consent
2. Baseline questionnaire for P5 participants
Knowledge of basic genetics
Perceived risk
3. Genotyping and imputation
3177 P5 participants with quality controlled genetic data of >17M variants
4. Evaluation of PRS and lifestyle risk factors in the FINRISK Study
Selection of T2D PRS
PRS calculation
Time-to-event analysis - added value of PRS
Risk estimates ready for implementation in 10-year risk calculation in P5 Study
5. Estimating the future risk of disease for P5 Study participants
PRS calculation
Summary statistics from the selected time-to-event model
6. Risk information through MyP5 web portal
7. Randomisation into two groups (RCT)
Group 1, N=1587
Group 2, N=1590
Questionnaire on perceived risk and health
Return of 10-year risk for T2D
Risk based on PRS & lifestyle factors
Doctor´s note
Risk based on lifestyle factors
Doctor´s note
Questionnaire on perceived risk and health
After 2mo: 10-year risk for T2D
Risk based on PRS & lifestyle factors
Doctor´s note
Questionnaire on perceived risk and health
